# Rapid Antiretroviral Therapy (ART) Initiation at a Community-Based Clinic in Jackson, MS

**DOI:** 10.1186/s12981-020-00319-7

**Published:** 2020-10-08

**Authors:** Courtney E. Sims Gomillia, Kandis V. Backus, James B. Brock, Sandra C. Melvin, Jason J. Parham, Leandro A. Mena

**Affiliations:** 1 Department of Population Health Science, John D. Bower School of Population Health, University of Mississippi Medical Center, 2500 N State Street, Jackson, MS 39216 USA; 2 Division of Infectious Diseases, School of Medicine, University of Mississippi Medical Center, Jackson, MS USA; 3 Open Arms Healthcare Center, Jackson, MS USA

**Keywords:** Rapid ART, Immediate ART, HIV treatment, Viral suppression

## Abstract

**Background:**

Rapid antiretroviral therapy (ART), ideally initiated within twenty-four hours of diagnosis, may be crucial in efforts to increase virologic suppression and reduce HIV transmission. Recent studies, including demonstration projects in large metropolitan areas such as Atlanta, Georgia; New Orleans, Louisiana; San Francisco, California; and Washington D.C., have demonstrated that rapid ART initiation is a novel tool for expediting viral suppression in clinical settings. Here we present an evaluation of the impact of a rapid ART initiation program in a community-based clinic in Jackson, MS.

**Methods:**

We conducted a retrospective chart review of patients who were diagnosed with HIV at Open Arms Healthcare Center or were linked to the clinic for HIV care by the Mississippi State Department of Health Disease Intervention Specialists from January 1, 2016 to December 31, 2018. Initial viral load, CD4+ T cell count, issuance of an electronic prescription (e-script), subsequent viral loads until suppressed and patient demographics were collected for each individual seen in clinic during the review period. Viral suppression was defined as a viral load less than 200 copies/mL. Rapid ART initiation was defined as receiving an e-script for antiretrovirals within seven days of diagnosis.

**Results:**

Between January 1, 2016 and December 31, 2018, 70 individuals were diagnosed with HIV and presented to Open Arms Healthcare Center, of which 63 (90%) completed an initial HIV counseling visit. Twenty-seven percent of patients were provided with an e-script for ART within 7 days of diagnosis. The median time to linkage to care for this sample was 12 days and 5.5 days for rapid ART starters (p < 0.001). Median time from diagnosis to viral suppression was 55 days for rapid ART starters (p = 0.03), a 22 day decrease from standard time to viral suppression.

**Conclusion:**

Our results provide a similar level of evidence that rapid ART initiation is effective in decreasing time to viral suppression. Evidence from this evaluation supports the use of rapid ART initiation after an initial HIV diagnosis, including same-day treatment.

## Background

Rapid antiretroviral therapy (ART) is a novel strategy to reduce HIV transmission and shorten time to virologic suppression. Rapid ART has been adopted in many cities throughout the United States and typically involves the provision of antiretroviral medications within 7 days of a new diagnosis. Rapid ART, ideally within 24-h of diagnosis, is proving to be crucial in efforts to speed virologic suppression and reduce HIV transmission [1–7]. Our national plan: “Ending the HIV Epidemic: A Plan for America” aims to improve quality of life for people living with HIV (PLWH) through early diagnosis and rapid and effective treatment to achieve and maintain viral suppression [8, 9]. Recommendations that ART should be initiated within 7 days of HIV diagnosis have been adopted by the World Health Organization (WHO) and International AIDS Society-USA (IAS-USA) [1, 3]. The Department of Health and Human Services (DHHS) also recommends the immediate initiation of ART therapy due to the significant increase in viral suppression amongst patients from the START and TEMPRANO randomized trials who began immediate ART or initiated therapy within 7 days [10].

Recent trials have demonstrated the benefits of starting ART as soon as possible following HIV diagnosis [1, 2, 4, 11]. Findings from the HPTN 052 trial informed the treatment as prevention strategy to combat HIV infection [12]. Evidence provided by the PARTNER and Opposites Attract prospective, observational studies support that the risk of HIV transmission in both gay and heterosexual couples through condomless sex when HIV viral load is undetectable is effectively zero [13, 14]. Moreover, earlier linkage to care and treatment has proven to improve patient outcomes and decrease AIDS progression and mortality [15].

Recent demonstration projects in large metropolitan areas such as Atlanta (ATL), New Orleans (NO), San Francisco (SF) and Washington D.C. (DC) have demonstrated that rapid ART initiation is a promising strategy for earlier virologic suppression in clinical settings [6, 7, 16]. Each of these cities benefitted from citywide and statewide initiatives to end HIV that promote faster linkage, greater access to care, expedited clinic intake and increased fiscal resources for rapid initiation. Median time to virologic suppression following ART initiation in ATL, NO, and SF was 57 days, 30 days, and 43 days, respectively [1, 6, 7]. The Fast Forward (FF) pilot program in DC did not see a significant difference between time from diagnosis to viral suppression between individuals who were in the FF group compared to standard of care (SOC) (80.6% vs. 74.2%) [16]. However, this could be attributed to the wrap-around services (i.e. insurance navigation, phlebotomy, nursing intake, and medical assessment) already included in their existing SOC model.

Barriers including poverty, housing insecurities and lack of health insurance impact linkage to care and can delay initiation of ART. Other structural barriers to rapid ART initiation include the absence of Medicaid expansion in the state of Mississippi and the process of approval for ART medications through the AIDS Drug Assistance Program (ADAP). The latter requires that new HIV diagnoses be registered in the Enhanced HIV/AIDS Reporting System (eHARS) with documentation of CD4+ T cell count and HIV viral load results which may take up to 14 days to be reported. These requirements can interfere with same-day access to HIV medications, especially for underinsured or uninsured individuals.

Open Arms Healthcare Center (OAHCC) is a community-based clinic in Jackson, Mississippi offering primary care services with an emphasis on the healthcare needs of gender and sexual minority populations. OAHCC has provided holistic HIV care since 2014 and has been a regional innovator of HIV service delivery since its inception. In January 2016, OAHCC began offering same-day ART initiation for newly diagnosed patients, dependent on each individuals’ willingness to start. The purpose of this analysis was to evaluate the impact of rapid ART initiation in a community-based clinic that provides primary HIV care in a city with high incidence and prevalence of HIV. In this evaluation, we compared the outcomes (viral loads and CD4+ T cell counts) of individuals who initiated ART within 7 days of receiving an HIV diagnosis (rapid ART) with those who did not (non-rapid starters).

## Methods

### Study setting

This study was conducted at Open Arms Healthcare Center (OAHCC), a community-based organization (CBO) under the direction of My Brother’s Keeper, Incorporated. OAHCC was incepted in 2012 with a mission focused on providing a safe space that offers holistic healthcare to Lesbian, Gay, Bisexual and Transgender (LGBT) individuals. The first HIV clinic began in 2014 and has grown to more than 400 established patients.

### Study design

We conducted a retrospective review of medical records of individuals newly diagnosed with HIV who presented to OAHCC for HIV care and treatment from January 1, 2016 through December 31, 2018. Patients either were diagnosed with HIV at OAHCC or referred for linkage to HIV treatment services from the Mississippi State Department of Health (MSDH) Sexually Transmitted Disease (STD) Clinic by a Disease Intervention Specialist after being notified of their HIV diagnosis. Initial clinic visits at OAHCC included clinical evaluation (i.e. nursing intake and phlebotomy), HIV-related education and counseling, enrollment in the Ryan White Part B program and ADAP, if applicable, and baseline laboratory tests. Patients were prescribed medication on the same day of their initial clinical visit when possible, depending on their expressed willingness and medication coverage. Uninsured patients were given access to medications through available medication samples or access through drug manufacturer medication assistance programs. The following variables were abstracted from the medical records by OAHCC staff during and included demographics, date of HIV diagnosis, date of first visit to a provider at OAHCC, risk factor(s) for HIV acquisition, drug use, insurance status and date of and results for initial and subsequent HIV viral load and CD4+ T cell counts.

Rapid ART starters were defined as individuals who started ART within seven days of an initial HIV diagnosis; all others were classified as non-rapid starters. Manufacturer-funded drug assistance programs were used to provide immediate access to ART pending commercial insurance or ADAP approval at no additional cost to patients. Primary statistical analyses compared time from ART initiation to viral suppression amongst rapid ART starters and non-rapid starters. Secondary analyses were used to determine differences in time from diagnosis to viral suppression between the two groups as well as well as the effect time from diagnosis to treatment initiation had on viral suppression.

#### Study measures and analysis

We compared outcomes data per year (2016–2018) and between groups. Viral suppression was defined as HIV RNA < 200 copies/mL. Date of ART initiation was measured using the date patients received their first e-script. Continuous variables were compared using Mann–Whitney test. Categorical variables were compared using Fisher’s exact test. Analyses were conducted using STATA SE 16.0 (Stata Corp., College Station, TX). This study was reviewed and approved by University of Mississippi Medical Center’s Institutional Review Board (IRB).

## Results

During the evaluation time period, 70 individuals newly diagnosed with HIV were evaluated for treatment at OAHCC. Sixty-three patients were included in the analysis. Individuals who did not have a detectable viral load at their initial HIV care visit were excluded (n = 2). Five patients were excluded from the analysis because they transferred care immediately following diagnosis (n = 2) or they did not return for their initial visit for HIV care (n = 3). Of the patients who met the criteria for analysis, 27% were rapid ART starters.

Demographic and behavioral characteristics of the sample population are shown in Table 1. Majority of the sample were men (87%), identified as non-heterosexual (75%) and were Black/African American (91%). The median age was 27-years old (IQR, 20–61). Fifty-seven percent of patients (36/63) were uninsured and received Ryan White-funded HIV services; only 31% had commercial health insurance coverage. Mean baseline viral load was 337,280 (± 134, 1230) copies/mL and the mean CD4+ T cell count was 446 (± 290.25) cells/μl. Initial CD4+ T cell counts were found to be statistically significant in rapid starters compared to non-rapid starters (p = 0.009). There were no statistical differences for age, race, sex, sexual orientation, drug use or health insurance status between rapid ART starters and non-rapid starters.

**Table 1:** Demographic and clinical characteristics of patients newly diagnosed with HIV treated at OAHCC, January 1, 2016–December 31, 2018

	All patients, *n* (%)	Rapid-ART group, *n* (%)	Non-Rapid ART group, *n* (%)	*P*-value for comparison of rapid versus non-rapid groups (α = 0.05)
Number	63	16	47	
Age (in years)				
Median Age (range)	27 (20–61)	29.5 (21–53)	27 (20–61)	0.38
Sex (gender when available)				0.50
Male	53 (84%)	14 (88%)	39 (83%)	
Female	8 (13%)	2 (13%)	6 (13%)	
Transgender female	2 (3%)	0 (0)	2 (4%)	
Race/ethnicity				0.32
Black/African American	57 (91%)	16 (100%)	41 (87%)	
Hispanic/latinx	3 (4.8%)	0 (0)	3 (6%)	
White	3 (4.8%)	0 (0)	3 (6%)	
Sexual orientation				0.35
Gay/homosexual	35 (56%)	11 (69%)	24 (51%)	
Heterosexual	16 (25%)	2 (13%)	14 (30%)	
Bisexual	12 (19%)	3 (19%)	9 (19%)	
Insurance				0.45
Uninsured	36 (57%)	8 (50%)	28 (60%)	
Commercial	20 (32%)	7 (44%)	13 (28%)	
Government Ins. (medicaid/medicare)	7 (11%)	1 (6%)	6 (12%)	
Substance use				0.84
Marijuana	14 (22%)	3 (18%)	11 (65%)	
Other (cocaine, opioids, etc.)	3 (5%)	1 (5%)	2 (12%)	
Baseline CD4+, HIV RNA (mean, ± SD)				
CD4+ T cell count (cells/μl)	446 (± 290.25)	603 (± 323.74)	393 (± 260.64)	0.009
HIV RNA (copies/mL)	49,768 (130–10 million)	22,800 (500–10 million)	60,199 (130–3.8 million)	0.076

Unadjusted clinical milestones per year are described in Table 2. Of the 20 individuals diagnosed with HIV infection in 2016, only two were rapid ART starters. Seventy percent (14/20) of patients were linked to care with a provider within 30 days. In 2017, 26 patients were diagnosed with HIV, 96% (25/26) were linked to care within 30 days, and 35% (9/26) started ART within 7 days. Seventeen patients were diagnosed with HIV in 2018, of which 88% (15/17) were linked to care within 30 days and 29% (5/17) were rapid ART starters. The median time to linkage for the entire sample was 12 days and was significantly shorter for rapid ART starters compared to non-rapid starters (5.5 days vs 15 days; p < 0.001).

**Table 2:** Unadjusted time from diagnosis to clinical milestones among patients newly diagnosed with HIV treated at OAHCC (n = 63) by year, 2016–2018

	2016	2017	2018
Outcomes			
Diagnosed, *n*	20	26	17
Started ART, *n *(%)	20 (100%)	26 (100%)	17 (100%)
Met rapid definition, *n *(%)	2(10%)	9 (35%)	5 (29%)
% In care ≤ 1 year	18(90%)	26 (100%)	16 (94%)
Diagnosis to care entry, median number of days (range)	17.5 (0–596)	12 (1–31)	9 (0–293)
First care visit to ART start, median number of days (range)	0 (0–13)	0 (0–24)	0 (0–81)
ART start to first HIV RNA < 200 cells/mL	39 (21–145)	47.5 (21–240)	47 (0–212)
Diagnosis to HIV RNA < 200 cells/mL	71.5 (34–644)	66.5 (21–252)	77 (6–322)

Group comparison of virologic outcomes are presented in Table 3. Median time from initial HIV diagnosis to ART start was 5.5 days for rapid ART initiators (vs. 20 days; p < 0.001). The median time from diagnosis to viral suppression decreased from 77 days in non-rapid starters to 55 days in rapid ART starters (p = 0.03). Time from ART start to viral suppression was not statistically different between rapid starters and non-rapid starters (50 vs. 46 days; p = 0.35).

**Table 3:** Unadjusted virologic outcomes of patients receiving HIV care at OAHCC by Rapid-ART Status, 2016–2018

	Rapid ART initiators (received a prescription ≤ 7 days of diagnosis), (IQR)	Non-rapid initiators (received a prescription > 7 days of diagnosis), (IQR)	*P*-value (α = 0.05)
Outcomes			
Diagnosed, *n*	16	47	
Diagnosis to care entry, median (range)	5.5 (0–7)	15 (0–596)	< 0.001
First care visit to ART start, median (range)	5.5 (0–7)	20 (8–608)	< 0.001
ART start to HIV RNA < 200 cells/mL	50 (21–172)	46 (21–240)	0.35
Diagnosis to HIV RNA < 200 cells/mL	55 (26–179)	75 (6–644)	0.03

## Discussion

Our findings provide evidence of successful implementation and delivery of a rapid ART program in community-based clinic in the Deep South. Acceptance of rapid ART was high and initiation proved to be feasible despite existing structural barriers. Of 63 individuals diagnosed with HIV from 2016 to 2018, only 5 individuals (8%) had an initial CD4+ T cell count < 200 cells/μl, indicating more recent HIV infections than those who may have been diagnosed previously. As a result of this program, individuals who were rapid ART starters achieved faster linkage to care and significant decreases in the time from the initial care visit to ART start as well as the time from initial diagnosis to viral suppression. This is consistent with a review of the literature that includes meta-analyses, prospective, observational and randomized studies.

To our knowledge, this is the first paper to report on the experience of rapid ART initiation in Mississippi. Our evaluation indicates that this demonstration project supports the use of rapid ART as a feasible approach in places that lack local government-mandated and funded initiatives. Rapid ART in this community-based clinic resulted in shorter times to linkage to care with an HIV provider and faster access to ART prescriptions. Rapid ART also serves as a tangible resource for addressing the Ending the HIV Epidemic initiative in the U.S.

Benefits achieved by rapid ART initiation were first examined in the CASCADE Randomized Clinical Trial conducted in sub-Saharan Africa. Viral suppression was achieved by 50.4% of same day starters versus 34.3% in the SOC group [4]. Linkage to care was also higher (68%) for same day starters compared to those in SOC (43.1%). This study supports the use of rapid ART initiation in high-risk patient settings. In an unblinded, randomized trial designed to test and evaluate the use of a rapid service delivery intervention findings suggest that those who benefitted from rapid ART the most were individuals who otherwise would not have initiated care [17]. While time from ART start to virologic suppression between groups in this evaluation was not significant, previous studies support that rapid ART initiation is beneficial to high-risk populations [2, 4, 18]. These non-significant findings may be attributed to the unknown period between patients being provided with a prescription for ART and actually starting their medication, due in part by delay caused by third party payers who require a viral load confirmation.

The success of rapid ART initiation at OAHCC is largely a result of the HIV diagnosis same-day referral partnership between OAHCC and MSDH. Despite the lack of ART starter packs that were provided to patients in previously mentioned demonstration projects, this program was still successful in satisfying ADAP requirements and lessening time from diagnosis to ART start by taking advantage of existing medication assistance programs when needed. The staff is comprised of three part-time physicians, one part-time and one full-time nurse practitioner, three case managers and several nurses dedicated to patient care. In addition, OAHCC is equipped with resources that address micro-barriers such as food insecurity and lack of transportation. Each individual who initiated ART therapy returned for their four week follow-up visit with a provider. This is a testament of the strong social support of OAHCC to maintain retention in care.

This evaluation has several limitations. Individuals who were classified as rapid ART starters may have had less barriers to care than individuals who were non-rapid starters, resulting in better outcomes for the rapid ART group. OAHCC lacks an on-site pharmacy; therefore, we were unable to verify that patients who received an ART prescription, filled the prescription and started taking antiretrovirals immediately. The ADAP process, which requires that individuals newly diagnosed with HIV be verified through the MSDH HIV reporting system and have initial HIV lab results before medication will be dispensed, contributed to a delay of 2–3 weeks between when the prescription was written and the patient received the medication. Moreover, the number of ART manufacturers that provide immediate access to medications while ADAP is pending is limited.

## Conclusions

Evidence from this evaluation supports the use of rapid ART initiation after an initial HIV diagnosis, including same-day therapy. This program provides evidence for the feasibility of Rapid ART initiation in hard-to-reach populations, including men who have sex with men (MSM). Future directions include implementing a government-funded initiative to help address the HIV disparities that exist throughout the state and to extend rapid initiation services to rural areas. Improvements to the ADAP medication process could increase medication access for newly diagnosed patients.

## Data Availability

Data availability must be on an “upon reasonable request” basis made to the corresponding author.

## Abbreviations

ART: Antiretroviral therapy
e-script: Electronic prescription
PLWH: People living with HIV
WHO: World Health Organization
IAS-USA: International AIDS Society USA
DHHS: Department of Health and Human Services
ATL: Atlanta
NO: New Orleans
SF: San Francisco
DC: Washington D.C.
FF: Fast forward
SOC: Standard of Care
ADAP: AIDS Drug Assistance Program
eHARS: Electronic HIV/AIDS Reporting System
OAHCC: Open Arms Healthcare Center
CBO: Community-based Organization
LGBT: Lesbian, Gay, Bisexual and Transgender
MSDH: Mississippi State Department of Health
STD: Sexually Transmitted Disease
IRB: Institutional Review Board
MSM: Men who have sex with men
